# Therapeutic strategies for gastric cancer targeting immune cells: Future directions

**DOI:** 10.3389/fimmu.2022.992762

**Published:** 2022-09-23

**Authors:** Yan Zhao, Yuansong Bai, Meili Shen, Yapeng Li

**Affiliations:** ^1^Department of Oncology and Hematology, China-Japan Union Hospital of Jilin University, Changchun, China; ^2^Department of Radiation Oncology, China-Japan Union Hospital of Jilin University, Changchun, China; ^3^The National and Local Joint Engineering Laboratory for Synthesis Technology of High Performance Polymer, College of Chemistry, Jilin University, Changchun, China

**Keywords:** gastric cancer, immunotherapy, immune cells, tumor microenvironment, treatment strategy

## Abstract

Gastric cancer (GC) is a malignancy with a high incidence and mortality, and the emergence of immunotherapy has brought survival benefits to GC patients. Compared with traditional therapy, immunotherapy has the advantages of durable response, long-term survival benefits, and lower toxicity. Therefore, targeted immune cells are the most promising therapeutic strategy in the field of oncology. In this review, we introduce the role and significance of each immune cell in the tumor microenvironment of GC and summarize the current landscape of immunotherapy in GC, which includes immune checkpoint inhibitors, adoptive cell therapy (ACT), dendritic cell (DC) vaccines, reduction of M2 tumor-associated macrophages (M2 TAMs), N2 tumor-associated neutrophils (N2 TANs), myeloid-derived suppressor cells (MDSCs), effector regulatory T cells (eT_regs_), and regulatory B cells (B_regs_) in the tumor microenvironment and reprogram TAMs and TANs into tumor killer cells. The most widely used immunotherapy strategies are the immune checkpoint inhibitor programmed cell death 1/programmed death-ligand 1 (PD-1/PD-L1) antibody, cytotoxic T lymphocyte–associated protein 4 (CTLA-4) antibody, and chimeric antigen receptor T (CAR-T) in ACT, and these therapeutic strategies have significant anti-tumor efficacy in solid tumors and hematological tumors. Targeting other immune cells provides a new direction for the immunotherapy of GC despite the relatively weak clinical data, which have been confirmed to restore or enhance anti-tumor immune function in preclinical studies and some treatment strategies have entered the clinical trial stage, and it is expected that more and more effective immune cell–based therapeutic methods will be developed and applied.

## Introduction

Cancer is the leading cause of death, and the incidence of gastric cancer (GC) remains high (1), most of which are advanced or metastatic at the time of diagnosis since a lack of reliable global screening strategies and specific symptoms (1); the chemotherapy-based comprehensive treatment is mainly the treatment method for this part of GC (2). In recent years, immunotherapy has received extensive attention from scholars, and some GC patients can benefit from this treatment. Compared with traditional chemotherapy, immunotherapy has the advantages of durable response, long-term survival benefits, lower toxicity, and active against brain metastases (3–6). At present, the widely used immunotherapy is immune checkpoint inhibitors (ICI) including programmed cell death 1/programmed death-ligand 1 (PD-1/PD-L1) antibody, cytotoxic T lymphocyte–associated protein 4 (CTLA-4) antibody, and chimeric antigen receptor T (CAR-T) in adoptive cell therapy (ACT). In addition, therapies targeting other immune cells have also demonstrated effectiveness in preclinical anti-tumor studies. Therefore, therapy targeting immune cells will become the most promising therapeutic strategy for GC.

**Figure 1:**
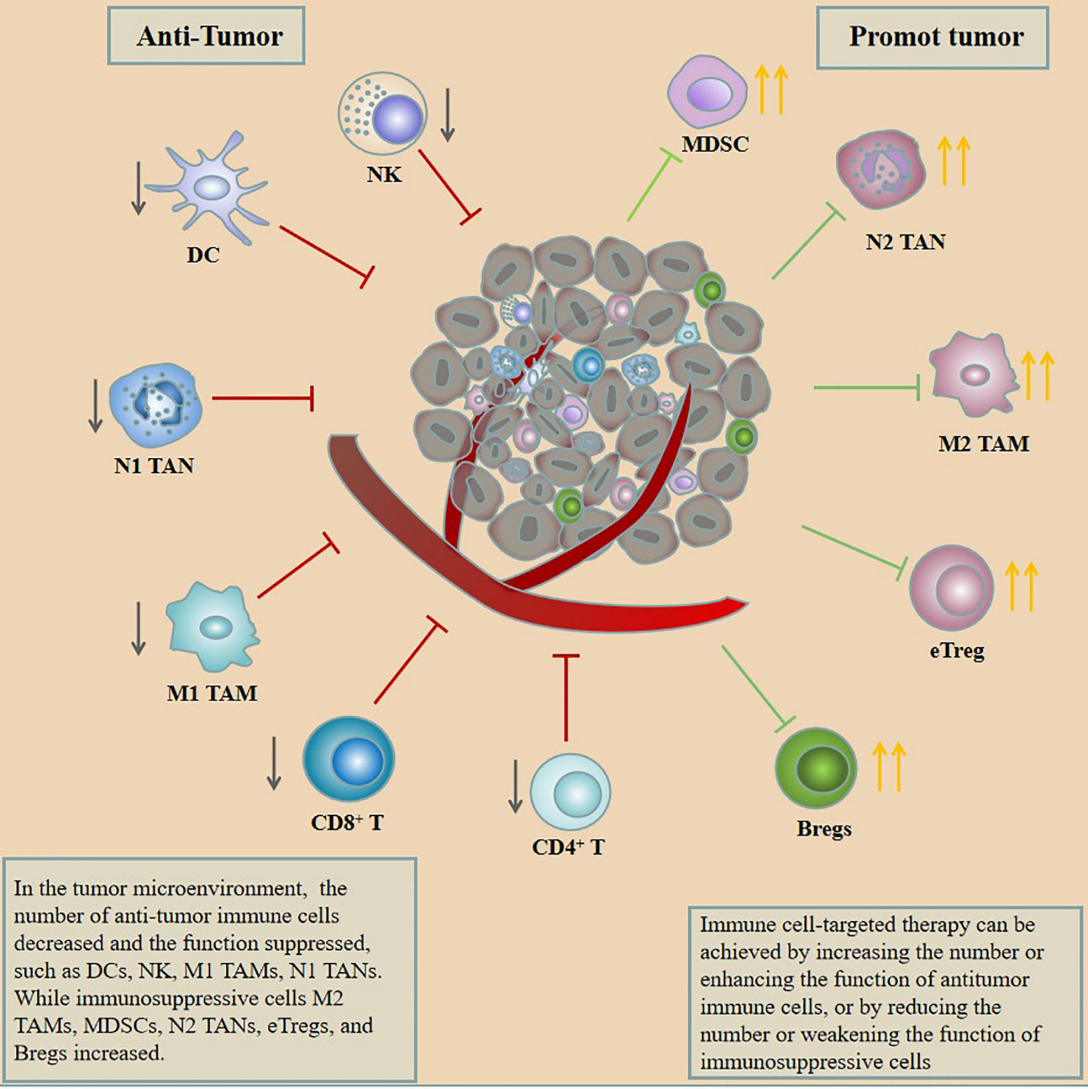
In the tumor microenvironment, the number of anti-tumor immune cells decreased and the function suppressed, such as DCs, NK, M1 TAMs, and N1 TANs, while immunosuppressive cells M2 TAMs, MDSCs, N2 TANs, eT_regs_, and B_regs_ increased. Immune cell–targeted therapy can be achieved by increasing the number or enhancing the function of anti-tumor immune cells or by reducing the number or weakening the function of immunosuppressive cells.

Immune cells are the target cells of immunotherapy which as part of the tumor microenvironment (TME) include dendritic cells (DCs), natural killer (NK) cells, tumor-associated macrophages (TAMs), tumor-associated neutrophils (TANs), myeloid-derived suppressor cells (MDSCs), T cells, and B cells (7). Initiating immune cells to kill tumors needs to go through 3 steps: 1. Neoantigens produced by the tumor are released and captured by DCs for processing. 2. DCs present the captured antigens on major histocompatibility complex (MHC) molecules to T cells, resulting in the priming and activation of effector T cells against the cancer-specific antigens. 3. Activated T cells migrate and infiltrate tumor tissue to destroy their targeted cancer cells (8). Tumor cells can evade recognition and clearance by the immune system through various mechanisms in the above three steps. Among them, M2 TAMs, N2 TANs, MDSCs, effector regulatory T cells (eT_regs_), regulatory B cells (B_regs_), and inhibitory targets on various immune cells play an important role in tumor immune escape, whereas the tumor-infiltrating DCs, NK, M1 TAMs, and N1 TANs are beneficial to anti-tumor immunity. In addition, anti-tumor immunity can be restored by increasing the number or enhancing the function of anti-tumor immune cells or by reducing the number of weakening the function of immunosuppressive cells (**Figure 1**). Based on this, a lot of trials have been carried out for targeting immune cells to treat tumors. This review introduces the impact of immune cells on the occurrence and development of GC from the perspective of immune cells in the tumor microenvironment of GC and summarizes the current strategies for targeting immune cells to treat GC.

## T cell–based immunotherapy

Targeted T-cell immunotherapy is the most studied and promising therapeutic approach in the field of GC. Numerous clinical trials have been conducted and some of these treatments have achieved remarkable results. T cells are part of the adaptive immune system, and cellular components are phagocytosed after tumor cell lysis and expressed on antigen-presenting cells (APCs) then exposing them to mature lymphocytes and leading to tumor suppression (9). Different subsets of T cells determine different tumor prognoses (10). Among which T-cell subsets that are beneficial to the prognosis and survival of GC include high levels of CD8^+^ and CD4^+^ T-cell infiltration (11), high CD45RO memory T cells (12, 13), and high T helper (T_H_)1/T_H_2 ratio (14). While the T-cell subsets associated with GC occurrence and progression are mainly tumor infiltrative forkhead box P3-positive (FOXP3^+^) T_regs_ (15, 16), FOXP3 is a transcription factor that is specifically expressed by natural T_regs_ (17). It is well known that HP is the main pathogenic factor of GC and studies have shown that the expression level of FOXP3^+^ T_regs_ in HP-infected patients is significantly higher than that in non-infected patients (18), and the number of FOXP3^+^ T_regs_ is significantly elevated in gastritis and GC in HP-infected patients (19). In addition, the expression level of FOXP3 T_regs_ is closely related to the occurrence and development of GC (20, 21). A significant increase in the proportion of T_regs_ has been reported in tumor-draining lymph nodes in patients with GC (22). In addition, highly expressed FOXP3^+^ T_regs_ in sentinel lymph nodes in GC could predict the metastasis to downstream lymph nodes (23). However, the correlation between T_regs_ infiltration and GC prognosis remains unclear, and there have been many contradictory results because of different T_regs_ markers, location distances, and intracellular interactions. As reported, a high FOXP3/CD4 ratio has been associated with poor prognosis in gastric cardia cancer (24). The intratumoral infiltration of FOXP3^+^ T_regs_ suggests a poor prognosis in patients after radical gastrectomy (21). Moreover, an increase of FOXP3^+^ T_regs_ infiltration in GC tissues and a high proportion of T_regs_/CD8^+^ T cells both predict a poor prognosis (25). However, there are some reports to the contrary, for example, patients with high FOXP3 T_regs_ cells have a good prognosis in the microsatellite unstable GC population (26). In gastric cardia cancer, stromal infiltration of T_regs_ may inhibit GC progression caused by chronic inflammation (27). The high expressions of epithelial-infiltrating CD8^+^ T cells and FOXP3^+^ T_regs_ in the GC microenvironment also predict a good prognosis; the authors suggested that the spatial distance between T_regs_ and CD8^+^ T cells determines their effect on prognosis, with an intervening distance of 30–110 μm predicting a favorable prognosis (28). In addition, the reason for these two contradictory reports may also be related to different T_regs_ subsets; some subsets may specifically mediate immunosuppression, such as ICOS T_regs_ cells that play a major suppressive role in the GC tumor microenvironment and which are mainly found in advanced GC patients (29). Therefore, distinguishing T_regs_ subsets is also valuable for predicting the prognosis of GC patients, and immunotherapies targeting certain T_regs_ subsets may lead to better efficacy. T cell–based immunotherapies include ICIs [PD-1/PD-L1, CTLA-4, lymphocyte activation gene 3 (LAG-3), T-cell immunoglobulin and mucin domain-3 (TIM-3), T-cell immunoreceptor with immunoglobulin, and ITIM domain (TIGIT)], ACTs [CAR-T, tumor-infiltrating lymphocyte (TIL), and cytokine-induced killer (CIK)], and T_regs_-targeted therapy strategy.

## PD-1/PD-L1

The anticancer activity of T cells depends on the detection of antigenic peptides present on the MHC by the T-cell receptor (TCR) (30) and the interaction between co-stimulatory signals and CD80/CD86 (B7 molecule) expressed on APCs and with cytokine interaction (31). Tumor cells can escape the killing of tumor cells by T cells through downregulating MHC and upregulating the expression of inhibitory receptors such as PD-L1, CTLA-4, LAG-3, TIM-3 and TIGIT (32–34), and blocking the binding of these inhibitory receptors to ligands can restore T-cell anti-tumor activity (35). PD-1/PD-L1 monoclonal antibodies (mAbs) demonstrate significant anti-tumor effect and prolong overall survival (OS) in various malignancies (36–38). PD-1 is an immunosuppressive receptor that is highly expressed on TILs (35), whereas PD-L1 as the main ligand of PD-1 is highly expressed on the surface of tumor cells (39). Tumor cells transmit negative regulatory signals to T cells through the interaction of PD-1 and PD-L1 thus inhibiting T cells activation and reducing cytokine secretion and promoting lymphocyte apoptosis, which was considered to be the main factors in promoting tumor immune escape (40), so antibodies that block PD-1 or PD-L1 can restore anti-tumor activity (41). More than 40% of GC patients express PD-L1 in gastric carcinoma tissues, and the expression of PD-L1 is significantly correlated to tumor size, invasion, lymph node metastasis (LNM), and survival time of patients (42).

Anti–PD-1/PD-L1 mAbs have demonstrated good efficacy in the treatment of GC. In the first-line treatment of GC, pembrolizumab combined with chemotherapy has a higher objective response rate (ORR) in people with a combined positive score (CPS) of ≥10, and the survival benefit of pembrolizumab monotherapy for GC was greater than that of chemotherapy in patients with microsatellite instability-high (MSI-H) (43). In the second-line treatment of gastric and esophagogastric junction adenocarcinoma, pembrolizumab improved OS compared with paclitaxel in patients with a CPS of ≥ 10, and, in the MSI-H subgroup, the OS significantly benefit; moreover, pembrolizumab had a better safety profile than paclitaxel (44). In the third-line treatment of GC, the ORR of pembrolizumab in the treatment of PD-L1–positive GC (CPS ≥1) patients was 15.5% (45), and, based on this study, the pembrolizumab was approved in the treatment of PD-L1–positive advanced GC by FDA. Nivolumab is another PD-1 inhibitor, which improved ORR, disease control rate (DCR), progression-free survival (PFS), and OS when compared with placebo in the third-line or later-line treatment of GC (46). Currently, nivolumab has been approved in China (47), Japan, South Korea, and other countries as a third- or later-line therapeutic option in heavily pretreated patients with unresectable advanced or recurrent gastric/gastroesophageal junction cancer (G/GEJ) cancer (48). In addition, nivolumab combined with chemotherapy [capecitabine plus oxaliplatin (CapeOx) or leucovorin calcium plus fluorouracil plus oxaliplatin (FOLFOX)] in the first-line treatment of metastatic G/GEJ cancer was compared with chemotherapy alone, combination therapy demonstrated PFS benefit in patients with CPS ≥ 1 and in all randomized patients, and statistical significance in patients with CPS ≥5 and the median OS (mOS) of combination therapy was longer than chemotherapy alone in this population. Thus, nivolumab combined with FOLFOX/CapeOx is recommended for late-stage GC with PD-L1 CPS of ≥5 (49). Several phase II studies have shown that combined therapy using anti-human epidermal growth factor receptor 2 (HER2) drugs combined with PD-1 antibody or antiangiogenic inhibitor combined with PD-1 antibody could be a potential treatment strategy in HER2-positive GC patients (50, 51). Such regimens are currently being investigated in stage III clinical trials. Sintilimab (anti–PD-1 mAb) plus CapeOx showed promising efficacy with encouraging pCR rate, significant downstaging effect, and good safety profile in neoadjuvant immunotherapy for GC. This combination regimen might present a new option for patients with locally advanced, resectable G/GEJ adenocarcinoma (52). In addition, the KEYNOTE-585 study is an ongoing phase III study conducted to evaluate the efficacy and safety of pembrolizumab plus chemotherapy compared with placebo plus chemotherapy as neoadjuvant/adjuvant treatment for localized G/GEJ cancer (53). A phase III study investigating the efficacy and safety of neoadjuvant-adjuvant anti–PD-L1 antibody durvalumab and 5-fluorouracil-leucovorin-oxaliplatin-docetaxel (FLOT) chemotherapy followed by adjuvant durvalumab monotherapy in patients with resectable G/GEJ cancer is also underway recruiting (54). Data from these clinical trials may lay the foundation for the use of immunologic drugs in neoadjuvant treatment of GC.

PD-1 and PD-L1 mAbs have an encouraging survival advantage in GC. Unfortunately, PD-1 and PD-L1 antibodies failed to improve OS and PFS in some trials. For example, the KEYNOTE-061 study showed that second-line treatment with pembrolizumab compared with paclitaxel did not significantly prolong OS in patients with PD-L1 CPS ≥ 1 (44). In KEYNOTE-062, pembrolizumab or pembrolizumab in combination with chemotherapy in first-line treatment of advanced GC, ICI monotherapy, or in combination with chemotherapy did not demonstrate significantly improved survival rates compared to chemotherapy (43). Although PD-1/PD-L1 mAbs have obtained positive results in some clinical trials, most of the benefited groups are only those with high PD-L1 CPS score, MSI-H, or high tumor mutational burden (TMB) (44), and it has also been reported that patients with Epstein–Barr virus (EBV)–positive tumors can respond significantly to anti–PD-1 antibody (55). Unfortunately, the abovementioned benefit population does not account for much of advanced GC (the clinical trial results mentioned above are summarized in **Table 1**).

**Table 1:** Results of major clinical trials of PD-1/PD-L1 and CTLA-4 immune checkpoint inhibitors in the treatment of gastric cancer.

NCT Trial name	Conditions	Interventions	Therapy Line	Key Outcome	Phase	References
**CheckMate 649**	HER2-negative G/GEJ/EA cancer	Nivo + CapeOx/FOLFOX vs. CapeOx/FOLFOX	First line	OS: 13.1 m vs. 11.1 m; PFS: 7.7 m vs. 6.05 m.	III	(49)
**KEYNOTE-062**	PD-L1 (CPS≥1) advanced G/GEJ cancer	Pem vs. Pem + FP/XP vs. FP/XP + placebo	First line	mOS: 10.6 m vs. 12.5 m vs. 11.1m; mPFS: 2.0 m vs. 6.9 m vs. 6.4 m.	III	(43)
**KEYNOTE-061**	Advanced G/GEJ cancer	Pem vs. PTX	Second line	mOS: 9.1 m vs. 8.3 m; mPFS: 1.5 m vs. 4.1 m.	III	(44)
**KEYNOTE-059**	Advanced G/GEJ cancer	Pem vs. Pem + FP/XP	First line	ORR: 25.8% vs. 60.0%.	II	(56)
**KEYNOTE -063**	(PD-L1)-positive (CPS≥1) G/GEJ cancer	Pem vs. PTX	Second line	mOS: 8 m vs. 8 m; mPFS: 2 m vs. 4 m; ORR: 13% vs. 19%.	III	(57)
**KEYNOTE-811**	HER2- positive G/GEJ cancer	Pem + trastuzumab + FP/CapeOx vs. trastuzumab + FP/CapeOx	First line	ORR: 74.4% vs. 51.9%	III	(58)
**ATTRACTION-2**	Unresectable advanced or recurrent G/GEJ cancer	Nivo vs. placebo	Third line and above	mOS: 5.26 m vs. 4.14 m	III	(46)
**ATTRACTION-4**	Unresectable, advanced, or recurrent HER2-negative GC/GEJ cancer	Nivo + SOX/CapeOx	First line	The ORR of Nivo + SOX: 57.1%; mPFS: 9.7 m. The ORR of Nivo + CapeOX: 76.5%, mPFS: 10.6 m.	II	(59)
NCT04065282	Locally advanced, resectable G/GEJ cancer	Sintilimab + CapeOx	Neo adjuvant	The R0 resection rate: 97.2%. The 1-year DFS and OS rates: 90.3% and 94.1%	II	(52)
**CheckMate-032**	Locally advanced or metastatic chemotherapy refractory G/GEJ/ esophageal cancer	Nivo vs. Nivo + ipi	Third line and above	ORR: 12% vs. 24%	II	(60)
**NCT02658214**	Advanced G/GEJ cancer	Durv + trem vs. Durv vs. trem	Second line and above	mOS: 9.2 m vs. 3.4 m vs. 7.7 m	Ib/II	(61)

G, Gastric; GEJ, gastroesophageal junction; EAC, esophageal adenocarcinoma; m, months; vs., versus; Nivo, nivolumab. Pem, pembrolizumab; CapeOx, capecitabine plus oxaliplatin; FOLFOX, leucovorin plus fluorouracil plus oxaliplatin; FP, fluorouracil plus  cisplatin; XP, oxaliplatin plus  cisplatin; PTX, paclitaxel; SOX, S-1 plus oxaliplatin; ipi, ipilimumab; trem, tremelimumab; Durv, durvalumab; OS, median overall survival; mPFS, median progression free survival.

## CTLA-4

CTLA-4 inhibits T-cell activity by binding to B7 on the surface of APCs (62), whereas anti–CTLA-4 antibodies can relieve the inhibition of T cells by specifically binding to CTLA-4. These antibodies mainly include ipilimumab and tremelimumab. Ipilimumab monotherapy showed 14% ORR in chemotherapy-progressed advanced GC in a phase I/II clinical trial (CheckMate-032) (63). However, ipilimumab did not have significant survival benefits as maintenance therapy after first-line chemotherapy in phase II clinical trial (64). Another anti–CTLA-4 antibody drug tremetimumab acquired 1.7-month PFS and 7.7-month OS in second-line treatment of G/GEJ cancer in IB/II clinical trial (61). Although no exciting anti-tumor activity was shown in all patients, some patients have observed durable anti-tumor activity, ipilimumab suggests that some patients may benefit from tremelimumab therapy (65). In addition, in the treatment regimen of anti–PD-1 combined with anti–CTLA-4, CheckMate-032 showed that the ORR of the combination therapy group (nivolumab combined with ipilimumab) in the treatment of esophagogastric cancer was higher than that of the nivolumab monotherapy group, and the benefit of combination therapy was more obvious in the PD-L1–positive and MSI-H subgroups (60) (summary in **Table 1**).

## LAG-3

Antagonizing LAG-3 can restore the anti-tumor activity of T cells (66). LAG-3 as an inhibitory receptor expressed on activated T cells can downregulate T-cell function and participate in T-cell exhaustion and tumor immune escape thus promoting tumor progression (67, 68), and this immunoglobulin superfamily gene was also expressed on NK, DCs, and B cells (66, 69). T_regs_ cells expressing LAG-3 can suppress tumor-specific T cells and produce high levels of the immunoregulatory cytokines interleukin (IL)-10 and transforming growth factor-beta (TGF-β) (70, 71). The LAG-3 inhibitor relatlimab has been shown to deplete leukemia cells and restore NK and T cell–mediated anti-tumor activity in chronic lymphocytic leukemia (72). So far, more than 10 LAG-3 blockers such as relatlimab (73), ieramilimab (74), and HLX26 (68) have entered clinical trials. In addition, multi-targeted ICI combination therapy targeting LAG-3 can improve anti-tumor efficacy because LAG-3 is frequently co-expressed with PD-1 on TILs (73). Anti–LAG-3 plus anti–PD-1 therapy prolonged PFS in patients with advanced melanoma compared with anti–LAG-3 and anti–PD-1 alone in a phase III melanoma trial (68). At present, several clinical trials targeting LAG-3 in the treatment of GC are being carried out (**Table 2**), which is expected to become the next immunotherapy target for the treatment of GC.

**Table 2:** Clinical trials of targeting LAG-3 in gastric cancer.

Serial number	ClinicalTrials.gov Identifier	Conditions	Interventions	Phase	Status
**1**	NCT03662659	Gastric Cancer et al	Relatlimab/Nivolumab/Chemotherapy	II	Active, not recruiting
**2**	NCT03044613	Gastric Cancer et al	Nivolumab/Relatlimab/Carboplatin/Paclitaxel	I	Active, not recruiting
**3**	NCT04082364	Gastric Cancer	Margetuximab/retifanlimab/tebotelimab/trastuzumab/chemotherapy	II/III	Active, not recruiting
**4**	NCT04178460	Gastric Cancer et al	Niraparib combined with MGD013	I	Recruiting
**5**	NCT05144698	Gastric Cancer et al	RAPA-201	II	Recruiting
**6**	NCT03538028	Gastric Cancer et al	INCAGN02385	I	Completed
**7**	NCT03849469	Gastric Cancer et al	XmAb^®^22841/Pembrolizumab	I	Recruiting

## TIM-3

Targeting TIM-3 is another strategy based on T-cell immunotherapy. TIM-3 is an important tumor immune checkpoint expressed on a variety of immune cells including effector T cells, monocytes, NK, and DCs (75, 76), which can inhibit innate and T-cell immune response (77, 78), participate in immune escape (79), and promote immune tolerance (80). Blocking TIM-3 pathway can positively regulate innate and adaptive immunity, alleviate T-cell depletion, and increase the secretion of interferon-γ (IFN-γ) by NK and T cells (75, 81). TIM-3 and its ligands are highly expressed in various solid tumors such as GC, and its overexpression was associated with the aggressiveness, late stage, and poor prognosis of malignant tumors (75, 82–84). Therefore, TIM-3 can be used as a prognostic biomarker for various solid tumors. Three polymorphisms (−574G/T, −882C/T, and −1516G/T) within the TIM-3 gene were found to be significantly associated with GC in evaluating the association between TIM-3 gene variants and GC development, among which the −1516 polymorphism genotype was associated with distant metastasis of GC (85). The limited clinical efficacy of anti–TIM-3 antibody (LY3321367) led to the early termination of the study despite which has good pharmacokinetics/pharmacodynamics in the treatment of advanced solid tumors (86). However, it did not block the exploration of this target. Currently, there are two clinical studies on anti–TIM-3 treatment of GC, and we look forward to the announcement of positive clinical data. At the same time, the strategy of multi-target–combined blockade was also being explored in therapy. Dual blockade of TIM-3 and PD-1 seems to improve the anti-tumor ability of T cells because blocking PD-1 can upregulate the expression of TIM-3 (87). Facts also proved this point of view, a clinical trial of TIM-3 antibody (sabatolimab) combined with PD-1 antibody (spartalizumab) showed anti-tumor activity and well tolerated in the treatment of advanced solid tumors (88). However, there are also studies that reported a bispecific antibody (bsAb) against TIM-3 and PD-L1 resulting in early study termination in the treatment of advanced solid tumors because all patients developed anti-drug antibodies (89). Therefore, strategies for targeting TIM-3 and in combination with other treatments require further research and improvement.

## TIGIT

TIGIT is an emerging immune checkpoint expressed on tumor-infiltrating CD8^+^ T cells, T_H_, T_regs_, and NK cells in various solid tumors including GC (90), which is associated with poor prognosis in various malignancies (91). Binding of TIGIT to CD155 ligand overexpressed on tumor cells led to immune escape while also suppressing innate and adaptive immunity, including inhibition of NK-mediated cytotoxicity and IFN-γ production, inhibition of DCs produces IL-12, inhibition of CD4^+^ T, CD8^+^ T cells proliferation, and induction of IL-10 production (92). Based on the negative regulatory effect of TIGIT on tumor immunity, currently, there are a variety of anti-TIGIT mAbs as monotherapy or combined with PD-1/PD-L1 antibody or combined with chemotherapy to treat tumors. Clinical data showed that anti-TIGIT antibody tiragolumab plus anti–PD-L1 antibody atezolizumab improved overall response and PFS and was well tolerated compared with anti–PD-L1 monotherapy in patients with PD-L1–positive metastatic NSCLC (93, 94). A phase II clinical trial (NCT04933227) in GC is underway to explore the efficacy and safety of atezolizumab in combination with tiragolumab and chemotherapy in the first-line treatment of HER2-negative recurrent or metastatic G/GEJ cancer.

## CAR-T

The ACT is T lymphocytes in peripheral blood or tumor-infiltrating is isolated and activated, expanded or genetically modified *in vitro*, and then infused back into the patient’s body. These treated T lymphocytes have potent anticancer activity (95). A meta-analysis showed that ACT treatment of GC could significantly improve PFS and prolong OS (96). ACT includes CAR-T (97), TIL ACT (98), and CIK ACT (97). CAR-T therapy is a promising therapeutic strategy in the field of cancer because it can cause target cell death and does not require activation of APCs (99, 100), which has an obvious curative effect in the treatment of hematological tumors (101). In the strategy of CAR-T treatment of GC, multiple targets have been proved to be effective in the treatment of xenograft mouse GC model, such as mesothelin (MSLN), prostate stem cell antigen (PSCA), B7 homology 3 (B7H3) protein, and NK group 2 member D (NKG2D) (102–105). In addition, CAR-T targeting carcinoembryonic antigen (CEA), HER2, CLDN18.2, FOLR1, c-Met, CD133, and CDH17 had a significant anti-tumor effect in the corresponding target-positive GC mouse model and the tumor appeared partially or even complete regression (106–112). CAR-T cells targeting ICAM-1 can effectively eliminate tumors developing in the lungs in animal models of GC (113). Modified CAR-T on the basis of the original target can play a role in increasing the efficacy, such as EGFR-CAR-T that secretes PD-1 scFv, c-Met CAR-T that adds PD-1/CD28 chimeric switch receptor (CSR), and bispecific human trophoblast cell surface antigen 2/PD-L1 CAR-T, which all have been proven to increase the efficacy of the original CAR-T therapy in mouse GC models (114–116). Up to now, there are as many as 30 clinical trials of CAR-T in the treatment of GC. Among them, the interim results of the phase I clinical trial of CLDN18.2-targeted CAR-T cells (CT041) in GC showed that CT041 treatment of CLDN18.2-positive GC patients had 57.1% ORR and 75.0% DCR; the 6-month OS rate was 81.2% and the safety was acceptable (117).

## TIL ACT

TIL ACT is usually isolated from the fresh tumor tissue of the patient and then returned to the patient after activation and expansion *in vitro*. GC TILs can be obtained from primary tumors (PTs), metastatic lymph nodes, and malignant ascites in GC patients, and TILs from different sites can recognize tumor cells in corresponding sites (118). TIL ACT has seen good efficacy in the treatment of GC. A clinical study showed that the survival time of the TIL treatment group was 50% longer than that of the chemotherapy group (119). Another report showed that intravenous injection of autologous TIL combined with recombinant IL -2 resulted in 13.0% tumor elimination (complete remission) and 21.7% tumor growth inhibition (partial response) of advanced GC patients (120). It has seen significant anti-tumor effects and good safety not only in the treatment of GC but also in various solid tumors (98). Therefore, it is a promising immunotherapy for the treatment of solid tumors. However, the wide clinical application of TIL ACT may be limited due to the high preparation cost, long production time, and the need for specialized facilities and personnel to produce TIL.

## CIK ACT

CIK has strong anti-tumor activity, MHC-independent, and antibody-dependent cytotoxicity (121). CIK can be produced by peripheral blood mononuclear cells (PBMCs) under the action of IFN-γ, IL-2, and anti-CD3 antibodies (122) and finally differentiate into CD3 CD56 CD8 NK-T cells. The cytotoxicity of CIK depends on the interaction of NKG2D receptors with NKG2D ligands and is mediated by perforin and can also regulate and enhance host cellular immune function by secreting cytokines and chemokines (123). In addition, CIK therapy can improve the survival rate of GC patients and can significantly improve the OS and disease-free survival (DFS) of patients with stage III GC when combined with chemotherapy (124). The 5-year OS and DFS were significantly prolonged after CIK treatment compared with the control group in patients with locally advanced GC (125). In addition, chemotherapy combined with CIK/DC-cytokine induced killer cell (DC-CIK) can significantly improve the OS rate, DFS rate, and T lymphocyte response in the treatment of postoperative patients with GC (105), and the survival was beneficial compared to chemotherapy alone (126).

## Therapies targeting T_regs_

**Figure 2:**
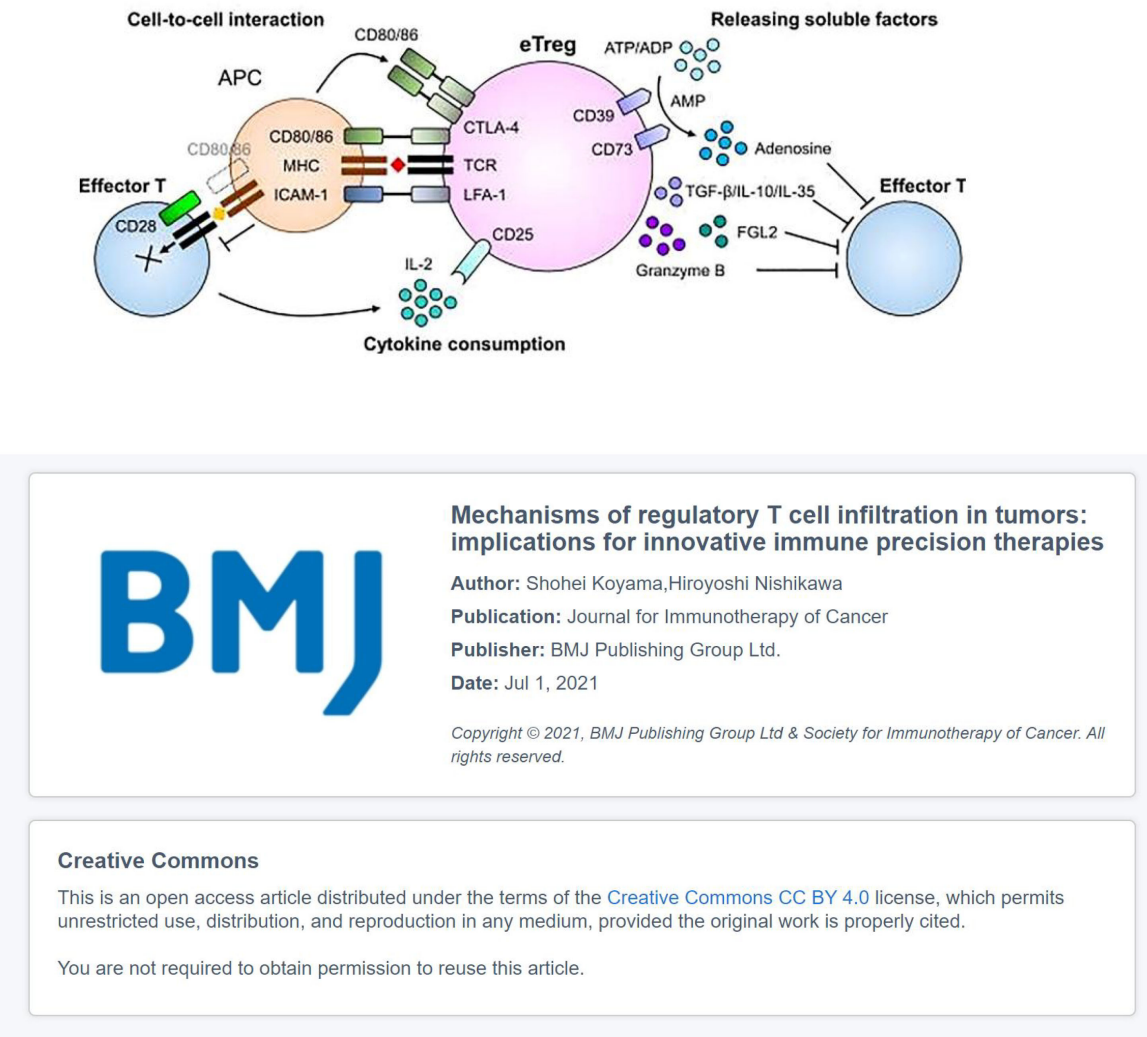
The immunosuppressive mechanisms of eT_regs_: The co-inhibitory receptor CTLA-4 in T_regs_ binds to CD80 and inhibits co-stimulatory signaling from APCs; T_regs_ can secrete inhibitory cytokines, including IL-10, TGF-β, and IL-35; T_regs_ can kill effector cells by granzyme and perforin and bind to the Fc fragment of IgG receptor IIB (FcγRIIB) in CD8^+^ T cells by secreting Fgl2, leading to their apoptosis. T_regs_ influence effector cell function: T_regs_ contain higher affinity receptor CD25 of IL-2, which compete with effector T cells to deplete IL-2, thereby inhibiting the growth of effector T cells; CD39 and CD73 expressed on the cell surface of T_regs_ act as ectonucleotidases that hydrolyze ATP or ADP to AMP and AMP to adenosine, respectively, thereby inhibiting effector T cells (131).

T_regs_ in TME can be classified into three types according to the expression of FOXP3 and CD45RA: non-T_regs_ (FOXP3^low^CD45RA^−^), naive T_regs_ (FOXP3^low^CD45RA^+^), and effector T_regs_ (eT_regs_) (FOXP3^high^CD45RA^−^). Non-T_regs_ cannot exert an inhibitory effect but can secrete pro-inflammatory cytokines. Naive T_regs_ are only weakly suppressive, whereas eT_regs_, which differentiate from naive T_regs_ after antigenic stimulation, possess the strong suppressive activity and stable function (127). T_regs_ express CTLA-4, PD-1, inducible T-cell costimulator (ICOS), glucocorticoid-induced TNFR-related protein (GITR), tumor necrosis factor receptor 4 (OX40), vascular endothelial growth factor receptor-2 (VEGFR2), chemokine receptor 4 (CCR4) and CCR8 receptors, which can mediate tumor immunosuppression (128); and participate in co-stimulatory receptors on the surface of APCs to modulate APC activity, leading to weaken or abrogated signals from APC to naive/effector cells. It can secrete immunosuppressive cytokines (IL-10, TGF-β, and IL-35) and immunosuppressive metabolites (tryptophan and adenosine), deplete the cytokine IL-2, and inhibit APC maturation (such as DCs) and tumor antigen-specific T-cell responses (128). eT_regs_ may also cause metabolic disruption to prevent naive/effector cell proliferation. Under certain circumstances, eT_regs_ could have a direct cytotoxic effect through the production of perforin/granzyme and induce apoptosis in effector cells. Dead T_regs_ can also rapidly convert ATP into adenosine in tumors, which then binds to receptors on the T-cell surface and affects T-cell function (129, 130), which in turn leads to the occurrence and progression of cancer (**Figure 2**).

Eliminating the immunosuppressive effect of T_regs_ is beneficial to restoring the tumor immune response, and the strategies can be achieved by reducing T_regs_, including direct strategies acting on T_regs_ surface molecules and indirect strategies to reduce T_regs_ through other ways. Direct strategies can be achieved by blocking surface molecules such as CD25, CTLA-4, PD-1, ICOS, GITR, OX40, and VEGFR2 on T_regs_. The CD25-blocking mAb daclizumab can result in the T_regs_ of patients significantly and a long-term decrease in the treatment of metastatic breast cancer patients (132). Loss or inhibition of CTLA-4 resulted in decreased T_regs_ function, and anti–CTLA-4 antibodies promoted anti-tumor activity by selectively reducing intratumoral T_regs_ (133). In addition, ICOS antibody KY1044, anti-GITR antibody TRX518, anti-VEGFR2 antibody ramucirumab (RAM), and chemotherapy drugs, such as low-dose cyclophosphamide, cyclosporine A, and tacrolimus, all can reduce T_regs_ in tumor patients (131, 134–136), while anti-OX40 antibody produced anti-tumor activity by blocking the inhibitory effect of T_regs_ (137). The strategies to indirectly reduce T_regs_ can be achieved by blocking the chemokine and/or cytokine axis, intracellular signaling pathways, and metabolites of T_regs_. T_regs_ can migrate to TME under the action of chemokines such as CCR4-CCL17/22, CCR8-CCL1, CCR10-CCL28, and CXCR3-CCL9/10/11 (128), so blocking chemokines and chemokine receptors can inhibit T_regs_ migration thus indirectly reduce T_regs_ in the TME. Anti-CCR4 mAb and anti-CCR8 mAb have been shown to selectively deplete tumor-infiltrating T_regs_ (138, 139). In addition, the tyrosine kinase inhibitor imatinib can selectively induce T_regs_ apoptosis by reducing the intensity of TCR signaling through the inhibition of lymphocyte-specific protein tyrosine kinase (LCK) (140). Specific inhibitors of phosphoinositide 3-kinase (PI3K) δ can also improve cancer immunotherapy by reducing the number of T_regs_ in the tumor microenvironment (141). At the same time, adjusting metabolites in the TME also affects T_regs_ numbers, such as targeting fatty acid uptake (e.g., inhibition of fatty acid transporter CD36 and lactate transporter monocarboxylate transporter 1), blocking fatty acid oxidation (carnitine palmitoyltransferase 1a inhibitor), and blocking fatty acid synthase (acetyl-CoA carboxylase inhibitor (5-(tetradecyloxy)-2-furoic acid)) all can inhibit T_regs_ proliferation and reduce T_regs_ in TME (131).

Strategies to reduce T_regs_ either directly or indirectly both can weaken the immunosuppressive effect of T_regs_. However, reducing T_regs_ in the TME may also reduce systemic T_regs_ and thereby increase the risk of immune-related adverse events (irAEs), such as autoimmune-related toxicities. Therefore, strategies should be used that selectively deplete eT_regs_ in the TME with little effect on systemic T_regs_ and other T_regs_ subtypes in order to ensure the safety and efficacy of T_regs_ cell–targeted therapy. Therefore, the T_regs_-targeted therapy strategy needs to be further improved.

The most widely used and the most complete data are PD-1/PD-L1 mAbs and CTLA-4 mAbs in ICIs despite having various strategies for T cell–based immunotherapy in the treatment of GC. Particularly, PD-1 mAbs have clinical data on the first, second, and third lines. Many studies have shown that PD-1 and PD-L1 mAbs have an encouraging survival advantage in GC, which have been approved in several countries for the treatment of advanced GC. Unfortunately, PD-1 and PD-L1 antibodies failed to improve OS and PFS in some trials, and the benefit population in positive clinical trial results is only those with high PD-L1 CPS score, MSH-H, or high TMB, but this group of people is not many. Multi-target blockade therapy may be the future treatment direction as the discovery of successive multiple immune checkpoint receptors. In addition to ICIs recommended by guidelines, CAR-T therapy in ACT has also seen high ORR and DCR in clinical trials and other ACTs also have good prospects. However, the wide clinical application of ACT is limited because the preparation process is cumbersome and its high cost of ACT. At the same time, selective removal of eT_regs_ cells in the TME is also the future direction because most current T_regs_-targeted therapies lack selectivity.

## DCs-based immunotherapy

The application of DCs in anti-tumor therapy mainly includes cancer vaccines and DC-CIK. Studies have been conducted combining DCs vaccination with chemotherapy (142), radiotherapy (143), targeted therapy (144), and other immunotherapies (145). DCs are used in cancer vaccines related to the anti-tumor mechanism of DCs. DCs are professional APCs that capture antigens released by tumor cells and present them to T cells in tumor-draining lymph nodes thus resulting in the generation of tumor-specific cytotoxic T lymphocytes (CTLs) (146). DCs can also stimulate NK and B cells to activate humoral immunity (146, 147). Studies showed that tumor-infiltrating DCs were associated with clinical stage, invasion, metastasis, and better prognosis in GC patients (148, 149). GC patients with many DCs invasion had lower LNM and lymphatic invasion, and also 5-year survival (78%) and OS higher than patients with little DC invasion (150, 151).

DC vaccines have shown OS benefits in solid tumors such as prostate cancer (152), melanoma (153), glioblastoma (154), and ovarian cancer (155). The studies showed that inactivated tumor cells (156), tumor lysates (153), tumor vesicles (157), synthetic tumor peptides (158), or synthetic tumor antigen mRNA (159) all can be used as DC vaccine–loaded antigens. Antigens that can be used as GC vaccines include melanoma-associated antigen (MAGE) A3, HER2 (p369) peptide, gastin-17 diphtheria toxoid (g17DT), URLC10 or VEGFR1 epitope, and heat shock protein GP96 (160). Up to now, there are five clinical trials of DC vaccines in the treatment of GC (**Table 3**).

**Table 3:** Clinical trials of DC vaccine in the treatment of gastric cancer.

Serial number	ClinicalTrials.gov Identifier	Conditions	Interventions	Phase	Status
**1**	NCT00004604	Gastric cancer et al. solid tumor	CEA RNA-pulsed DCs cancer vaccine	I	Completed
**2**	NCT00027534	Gastric cancer et al. solid tumor	Dendritic cells loaded with TRICOM-CEA (6D)	I	Completed
**3**	NCT04567069	Gastric cancer	DC vaccine	I/II	Recruiting
**4**	NCT04147078	Gastric cancer et al. solid tumor	DC vaccine	I	Recruiting
**5**	NCT03185429	Gastrointestinal solid tumor	TSA-DC vaccine	Not applicable	Unknown

Unfortunately, up to now, there are not many positive results of DC vaccines in the treatment of GC. Although phase I/II clinical trial has shown that Wilms tumor 1 (WT1)–targeted DC vaccine was a potential treatment in advanced cancer including GC, only three GC patients were included in the enrolled patients and only one GC patient was effective (161). Therefore, strategies to target multiple antigens have been explored in order to improve the efficacy of GC vaccines. For example, OTSGC-A24 achieved an impressive OS (5.7 months) in the treatment of advanced GC, which is a HLA-A*24:02 peptide-conjugated vaccine targeting FOXM1, DEPDC1, KIF20A, URLC10, and VEGFR1 (162). In addition, whole GC cells can also be fused with DCs to generate DC tumor hybrids, which have the advantage of combining the powerful antigen-presenting capacity of DCs with all antigens expressed by tumor cells (163, 164). At the same time, DC vaccines can also be combined with chemotherapy, radiotherapy, and ICIs to increase efficacy. Moreover, DC vaccines combined with neoadjuvant chemotherapy (NAC) showed that the combined treatment was safe and increased pathological complete remission (tpCR) in the treatment of HER2-negative breast cancer (165). DC vaccines combined with chemotherapy (carboplatin/pemetrexed) also had good efficacy and tolerability as the first-line drug therapy for patients with advanced non-squamous non–small cell lung cancer without oncogenic drivers (166). In addition, DC vaccines combined with radiotherapy can significantly inhibit tumor growth and improve survival rate, which has been confirmed in many tumor types such as melanoma and esophageal cancer (156, 167). DC vaccines combined with ICIs are also an effective treatment strategy, and preclinical studies had shown that DC vaccines combined with PD-1 inhibitors led to smaller tumor volume and better OS in the treatment of hepatocellular carcinoma (HCC) (145). In terms of toxic and side effects, the side effects of DC vaccines mainly include influenza-like symptoms, fever, and local reactions at the injection site, so it is safe for cancer patients. At present, more than 200 clinical trials have shown high immunogenicity and safety of DC vaccines (158).

Another method of DCs treatment of tumors is DC-CIK. Studies had shown that DC-CIK combined with chemotherapy was effective and tolerable in the treatment of non–small cell lung cancer, breast cancer, colorectal cancer, GC, and other solid tumors. DC-CIK combined with chemotherapy can enhance cellular immune function and inhibit tumor invasion and metastasis in the treatment of advanced non–small cell lung cancer (168). DC-CIK combined with capecitabine prolonged PFS in the treatment of patients with recurrent and metastatic triple-negative breast cancer (169). In addition, DC-CIK combined with adjuvant chemotherapy can significantly prolong the DFS of patients with postoperative colorectal cancer (170). In the treatment of GC, a meta-analysis showed that DC-CIK combined with chemotherapy can significantly improve the OS rate, DFS rate, and T lymphocyte reaction in patients after GC surgery (171). In addition, DC-CIK combined with S-1 and cisplatin had good PFS and OS in the treatment of advanced GC, and the combination therapy was safe and the toxicity was tolerable (172).

Tumor-infiltrating DCs are associated with a better prognosis in GC, but only a few mature DCs in the tumor microenvironment. DC-based immunotherapies such as cancer vaccines and DC-CIK have limited efficacy as a single treatment for cancer. Therefore, in order to increase the anti-tumor effect, it is very necessary to find the reasons for the low efficacy or combine it with other treatments to treat tumors.

## Immunotherapy targeting NK cells

NK cells are responsible for destroying tumor cells and preventing tumor initiation and progression. Activated NK cells can exert direct cytotoxicity through death receptor signaling, perforin, or release granzymes, which can also modulate other parts of the immune response by producing cytokines and chemokines (173). However, tumor cells can escape NK cell destruction by binding to inhibitory receptors expressed on the surface of NK cells. At the same time, the overproduction of TGF-β and other anti-inflammatory cytokines and chemokines in the tumor microenvironment can inhibit NK cell activation (174, 175); downregulate NK cell–activating receptors NKp30, NKp44, NKG2D, and CD16 and co-receptors NKp80 and DNAM-1; upregulate checkpoint receptors TIGIT, TIM-3, LAG-3, and PD-1; impair the expression and secretion of CD107; and secrete a variety of immunosuppressive factors (175, 176). Studies have shown that NK cell inhibitory receptor antibodies can restore NK cell activity. The widely used anti–PD-1 and anti–PD-L1 can enhance NK cell–mediated anti-tumor effects. The expression of PD-1 on NK cells interacting with PD-L1 on cancer cells can decrease the responses of NK cells, whereas blocking PD-1 and PD-L1 can increase NK cells *in vivo* and trigger strong NK cell responses and cytotoxicity in mouse tumor models (177, 178).

The treatment strategies based on NK cells for GC include NK cell adoptive therapy (such as autologous NK cell infusion, allogeneic NK cell infusion, and CAR-NK), blocking the inhibitory receptors expressed on NK cells, and increasing the activity of NK cells (such as increased activating receptors expression on NK cells, activation of NK cells by cytokines, and increased immune clearance of tumors by NK cells).

At present, NK cell adoptive therapies for GC have been carried out in more than 20 clinical trials. Five of 19 patients achieved complete hematologic remission in the clinical trial of haploidentical NK cell therapy for acute myeloid leukemia (AML) (179). However, no objective clinical response was observed in patients with melanoma treated with autologous NK cells activated *in vitro* (180), which suggests that it is more effective in donors with mismatched killer immunoglobulin receptor (KIR) ligand in NK cell adoptive therapy. In addition, NK cells can also be modified into CAR-NK, which is similar to CAR-T cell activity *in vivo* and safer than CAR-T cells (181, 182), and no strict HLA-matching requirements (183). Preclinical studies in the treatment of GC have shown that mesothelin-targeted CAR-NK cells can effectively eliminate GC cells in both subcutaneous and intraperitoneal tumors and significantly prolong the survival time of the mouse (184). In addition, studies on CAR-NK therapy targeting HER2, Mucin-1, EpCAM, or PMSA for GC are being carried out (184). CAR-NK therapy is becoming a promising treatment strategy in cancer immunotherapy based on the existing research data and less toxic side effects.

Increasing the anti-tumor activity of NK cells can be achieved by increasing the expression of activating receptors on the surface of NK cells, activating NK cells with cytokines, and increasing the immune clearance of tumors by NK cells. Retroviral transduction with DTCR (PD1-DAP10/NKG2D) can significantly increase the expression of NKG2D (an activating receptor on the surface of NK cells) on the surface of NK92 cells, thus enhancing cytotoxicity against GC cells SGC-7901, and DTCR-NK92 cells showed strong anti-tumor activity in the GC mouse model (185). In addition, the cytokines can activate NK cells and promote their proliferation to enhance anti-tumor activity, such as IL-2, IL-12, IL-15, and IL-18 (186–188). NK cells activated by IL-2 which combined with anti–PD-1 can inhibit tumor growth in xenograft GC models (189). In addition, IL-15 can increase the infiltration of NK cells in the tumor (190) and improve the survival rate in the treatment of GC liver metastasis–bearing mice (191). Primer of blood NK cells with recombinant human (rh)IL-12, rhIL-15, and rhIL-18 (12/15/18) can lead to memory-like NK cell differentiation and enhance tumor response (192). At the same time, increasing the immune clearance of NK cells against tumors can also play an anti-tumor effect. NK cells carry out immune clearance of tumors by releasing cytotoxic particles, antibody-dependent cell-mediated cytotoxicity (ADCC), and protein-activated target cell apoptotic systems synthesized on the cell surface [FasL and tumor necrosis factor-α (TNF-α)]. The mAbs trastuzumab (193), pertuzumab (194), cetuximab (195), rituximab (196), and anti-CD3 × anticancer bsAbs (197) all can play anti-tumor effect by enhancing ADCC ability and NK cell activity.

Blocking the expression of inhibitory receptors on NK cells can restore NK cell function, reverse NK cell depletion, increase NK cell cytotoxicity against tumors, and inhibit tumor growth. The efficacy increased when combined with other targeted drugs or ICIs (195, 198). The inhibitory receptors expressed on NK cells include KIR, leukocyte immunoglobulin-like receptor (LILR), killer lectin-like receptor (KLR) (173), inhibitory receptor composed of NKG2A and CD94, B7H3 protein receptor, sialic acid–binding immunoglobulin-like lectin (Siglecs), TIM-3, LAG-3, TIGIT, CD-47, etc. (199–207). Blocking these inhibitory receptors can restore the activity of NK cells. In addition, immunoglobulin-like transcript 2 (also known as LILRB1) can also inhibit the proliferation and cytotoxic activity of infiltrating NK cells in GC tumor tissues (208), so blocking this receptor can enhance the activation and proliferation of NK cells. The elimination of leukemia cells increased by activating NK cell cytotoxicity when combined with lenalidomide in chronic lymphocytic leukemia patients (209). Currently, several clinical trials are underway to block NK cell inhibitory receptors (210). In addition, blocking TGF-β1 signaling can prevent dysfunction of NK cells and thus restore their activity (211), and gene silencing of the PI3K catalytic subunit PI3KCB can enhance the lytic activity of NK cells against tumors (212).

NK cells are more cytotoxic to tumors, less immunogenic, have faster response, and do not need additional connections to activate receptors when compared with effector T cells. For example, the current hot spot treatment strategy CAR-NK is a promising treatment strategy for GC due to its unique recognition mechanism, strong cytotoxicity, clinical safety, and the ability to reduce the risk of allogeneic reactions. However, the application of CAR-NK cells is limited because the complex preparation and expensive and solid tumors have no immune-specific target antigen, a loss of tumor antigens, low persistence, and other factors. Another method of targeting NK cells to treat tumors is to restore and increase the anti-tumor activity of NK cells, and some effects have been seen in related studies but the efficacy is limited when used alone; combination therapy may be a strategy to solve this problem.

## TAMs-targeted therapy

TAMs are one of the most important components of the tumor microenvironment and are potential targets for cancer therapy. At present, the most studied is the “reprogramming” of TAMs from tumor support cells to tumor killer cells, that is, the reconversion of M2 TAMs to M1 TAMs. In addition, treatment strategies based on TAMs also include limiting monocyte recruitment and localization and CAR macrophage (CAR-M) therapy. Macrophages are divided into two subtypes: M1 or classically activated macrophages and M2 or alternatively activated macrophages. M1 TAMs have the inhibition effects of tumor and anti-angiogenic (213), whereas M2 TAMs can promote the occurrence and metastasis of tumor cells, inhibit the anti-tumor response mediated by T cells, and promote tumor progression and tumor angiogenesis (214, 215). In tumors, M2 macrophages are dominating in the tumor microenvironment as the tumor progresses (216). Chronic inflammation is a feature in GC tumor microenvironment, which is derived from infections such as *Helicobacter pylori*. These pathogens can impair the response of M1 TAMs, induce the state of M2-like, increase macrophage apoptosis, and promote disease progression (217). In addition, TAMs are related to the occurrence, development, and prognosis of GC, and CD204-positive (an M2-polarized macrophage receptor) TAMs are an important risk factor for a gastric adenoma to develop into adenocarcinoma (218). At the same time, the number of TAMs can predict the size and stage of GC in the GC tumor microenvironment (219) and involved in tumor invasion and metastasis (220). Furthermore, M2 TAMs were associated with poor prognosis and were an independent prognostic factor for GC (221).

The effect of phagocytosing tumor cells can increase by “Reprogramming” TAMs from tumor-supporting cells to tumor-killer cells, including the use of targeted antibodies (for example, targeting MФ surface receptors involved in immune response regulation and targeting the circulation cytokines/growth factors), gene therapy, small-molecule inhibitors, episomal vector delivery of nucleic acids, etc. Among them, the most studied therapeutic strategy is targeted antibodies, and the targets include colony-stimulating factor 1 (CSF-1)/CSF 1 receptor (CSF-1R), Toll-like receptors (TLR7, TLR8, and TLR9), histone deacetylase (HDAC), PI3Kγ, CD40, and CD47 (222). Neutralizing antibodies or small-molecule inhibitors of CSF-1/CSF-1R, CD40 antibodies, and TGF-β blockers all have been shown to reprogram M2 TAMs to M1 TAMs (222–224). Furthermore, TLR agonists can induce M1 polarization by increasing the release of pro-inflammatory mediators (225), and this therapy has shown promise in preclinical solid tumor models and in the clinic (226–228). The inhibition of HDAC or PI3Kγ exerted anti-tumor effects by downregulating M2 and upregulating M1 molecules (222). CD47 also affects TAMs polarization, and the anti-CD47 antibody increased the ability of macrophages to phagocytose tumor cells by blocking the interaction of CD47 with SIRPα on macrophages, which has been demonstrated in various preclinical models of solid tumors (229, 230). In addition, the suppression effect of macrophages can be abolished by inhibiting monocyte recruitment and localization to tumor tissue by targeting macrophage chemokines or their receptors (e.g., chemokine 2, chemokine 5, and CSF-1R). For example, blocking the CCL2/CCR2 axis can inhibit monocyte recruitment, TAM infiltration, and M2 polarization. Knocking down CCR2 or blocking CCL2/CCR2 signaling with CCR2 antagonists can inhibit tumor growth and metastasis, reduce postoperative recurrence, and improve survival (231). CCL5-CCR5 and CXCL12/CXCR4 also mediate TAMs recruitment and polarization, so blocking their mediated signaling was also a potential therapeutic strategy (232, 233). Macrophage recruitment was also promoted when CSF-1R binds to its ligand CSF-1 (234, 235), whereas the CSF-1R antagonists PLX 3397 or pexidartinib prevented the recruitment of monocytes from the circulation to cancerous tissues (236). In addition, drugs such as bisphosphonates also affect TAM infiltration and polarization. For example, zoledronic acid has been shown to modulate the tumor microenvironment by reducing TAM infiltration and polarization state (237, 238), which can also reduce angiogenesis by macrophage (239). At the same time, the efficacy of some chemotherapy drugs (such as trabetidine) may be related to the ability to kill TAMs (240). In addition, radiation therapy also affects TAMs as low-dose radiation therapy can reprogram TAMs to an anti-tumor phenotype (241).

CAR-M has also been developed and applied based on the good efficacy of CAR-T cells in hematological tumors (242). The CAR based on CD3ζ was highly active in human macrophages, which can drive phagocytosis and kill target tumor cells in a Syk-dependent manner (243). Studies have shown that CAR-M can transform M2 TAMs into M1 TAMs, upregulate the antigen presentation mechanism, recruit antigens and present them to T cells, express pro-inflammatory cytokines and chemokines, and resist the effects of immunosuppressive cytokines. CAR-M exhibited the effects of antigen-specific phagocytosis and tumor clearance *in vitro*. The tumor burden is reduced and OS is prolonged after infusion of CAR-M of human in mouse xenograft tumor models (243). At present, the treatment of CAR-M has entered the clinical trial stage and one of them is a study of CAR-M in the treatment of HER2-overexpressing solid tumors, including HER2-positive GC and other solid tumors. Another is a study of CAR-M in breast cancer. In addition, studies on the combination of CAR-M and T-cell checkpoint inhibitors are also ongoing based on the interaction between CAR-M and the adaptive immune system (243).

TAMs are the most abundant immune cells in the tumor microenvironment. Many strategies targeting TAMs to treat tumors have been carried out in preclinical studies and were proved effective, looking forward to the data of these therapeutic methods in clinical studies.

## Therapeutic strategies targeting MDSCs

**Figure 3:**
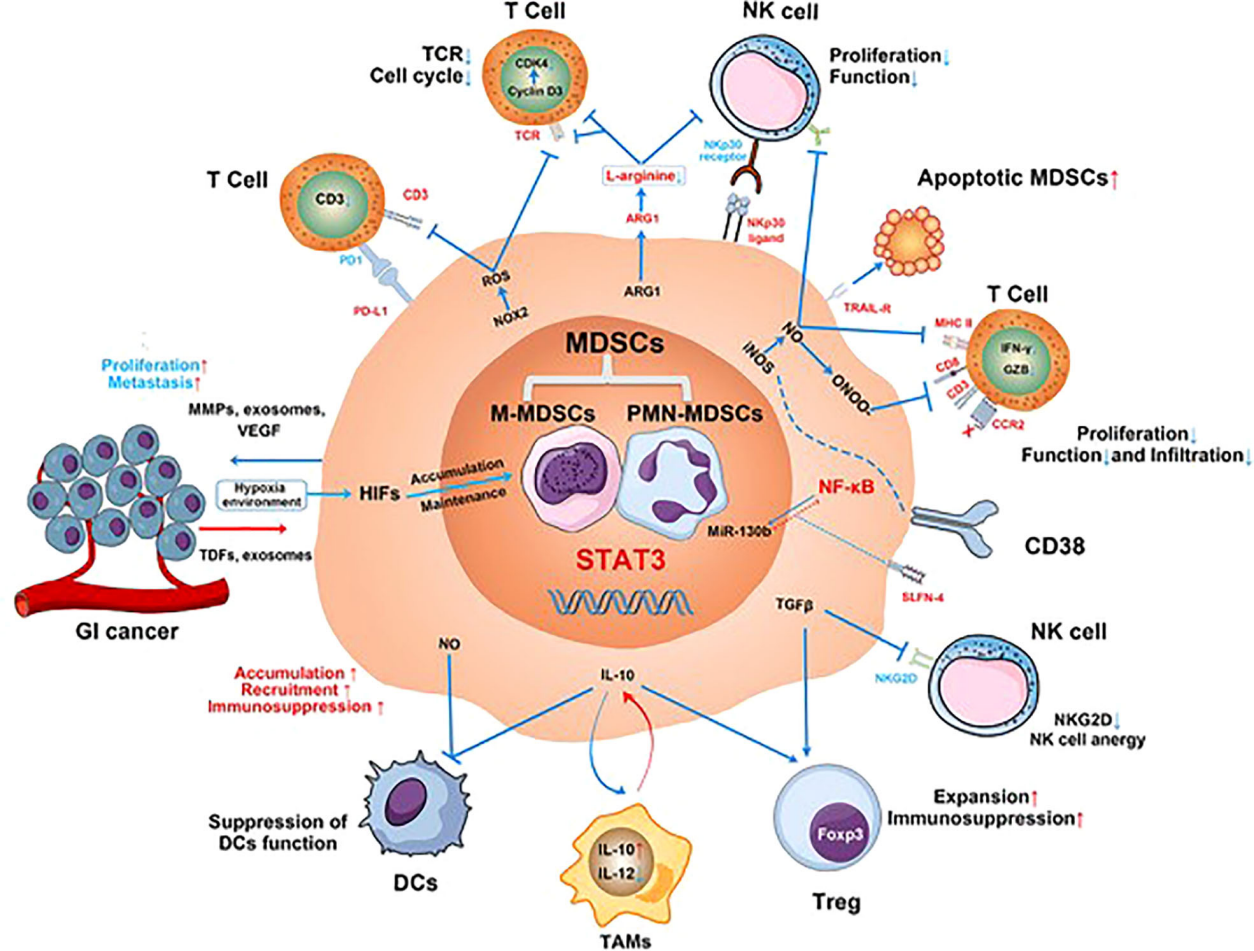
The mechanisms involved in MDSC-mediated immunosuppression in gastrointestinal (GI) cancer. MDSCs suppress proliferation and function of T cells and NK cells; reduce CD8^+^ T-cell infiltration; inhibit the function of DCs; inhibit the antigen presentation of DCs to CD4^+^ T cells; promote M2 macrophage differentiation; and promote T_regs_ expansion and immunosuppression. Additionally, the effect of ADCC function and anergy of NK cells is induced by the production of nitric oxide (NO) and the inhibition of NKG2D by TGF-β, respectively. MDSCs secrete matrix metalloproteinases (MMPs), exosomes, and vascular endothelial growth factors (VEGF) to promote GI cancer cell proliferation and metastasis (245).

MDSCs are a highly heterogeneous group of myeloid-derived cells whose most important function is immunosuppression (244). Inhibiting DC function and anti-tumor T-cell response, inducing NK cell apoptosis, promoting M2 TAMs differentiation, and increasing the number of T_regs_ (244, 245) (**Figure 3**) can promote the growth and metastasis of PTs (246, 247). Expansion of MDSCs was associated with resistance to treatment and poor prognosis in malignant tumors (246). In GC patients, the levels of MDSCs was associated with cancer stage and survival (248), such that higher levels of MDSCs were associated with later tumor stage and poor prognosis (249–251), as well as with higher mortality and risk of tumor recurrence and progression. In addition, patients with high MDSCs levels had significantly shorter OS than patients with low MDSC levels in patients with stage IV gastrointestinal cancer (252).

Eliminating the immunosuppression of MDSCs is beneficial for restoring anti-tumor immunity, and strategies include reducing circulating and tumor-infiltrating MDSCs and eliminating the immunosuppressive functions of MDSCs (244). The reduction of circulating and tumor-infiltrating MDSCs can be achieved with some chemotherapy drugs, targeted drugs, all-trans retinoic acid (ATRA), or by blocking the chemokine receptor on MDSCs. Low-dose chemotherapy drugs such as 5-fluorouracil (253), paclitaxel (254), gemcitabine (255), platinum (256), and Adriamycin (257) all have been shown to reduce MDSCs in cancer patients. 5-Fluorouracil combined with oxaliplatin reduced the number of MDSCs in the mouse model of GC (258). Epirubicin or docetaxel can reduce MDSCs and inhibit MDSCs function in GC patients and can also induce MDSCs apoptosis through mitogen-activated protein kinase (MAPK) and NF-kappa B signaling pathway (259). In addition, the targeted drugs apatinib and ATRA both can downregulate the proportion of MDSCs and reduce the number of MDSCs in blood circulation (260, 261). At the same time, ATRA can restore the accumulation of intratumoral MDSCs induced by anti-VEGFR2 (262). The number of MDSCs can be reduced by blocking the chemokine or cytokine receptors (e.g., CCR5, CSF-1R, CXCR1, and CXCR2) targeting MDSCs, which prevented myeloid marrow cells from trafficking into peripheral lymphoid organs or the tumor microenvironment (263–266). In addition, anti-CCR5 therapy also can reduce granulocyte-like MDSCs (G-MDSCs) and monocyte MDSCs (M-MDSCs) in peripheral and tumors of GC patients (267). At the same time, anti-CSF-1R can significantly reduce the ratio of MDSCs in tumor-infiltrating immune cells (268) and resulted in greater inhibition of tumor angiogenesis and tumor growth when combined with anti-VEGFR-2 antibodies (269). In addition, SX-682 (a small-molecule inhibitor of CXCR1 and CXCR2) can also inhibit the migration of MDSCs and eliminate the accumulation of MDSCs in tumor (270). The targeted drug Bruton’s tyrosine kinase ibrutinib had been confirmed to play an anti-tumor effect by reducing the recruitment and the number of MDSCs and inducing the maturation of MDSCs (271).

In addition, the growth of tumors can be driven by the interaction between immune cells and cancer stem cells (CSCs) in the tumor microenvironment. CSCs are the main cause of tumor metastasis, drug resistance, and recurrence (272). A study showed that GC tissue–derived mesenchymal stem cells (MSCs) can impair the anti-tumor immune response of PBMCs through disruption of the T_reg_/T_H_17 balance (273). MSCs can induce MDSCs in the tumor microenvironment (274), induce the generation of G-MDSCs, and modulate their activation, and can also coordinate MDSCs to transform the bone marrow (BM) microenvironment into an immunosuppressive environment (275). In addition, the metastasis of GC can be promoted through the inhibition of serine/threonine protein kinase 24 (STK24) expressed in normal and GC tissues, and this promotion was achieved by inducing overexpression of the GC stem cell marker CD44, enhancing CD11b^+^Ly6C^+^ MDSCs in the mouse spleen, and inducing their expansion (276). Infection with *H. pylori* [classified as a group 1 carcinogen by the World Health Organization (WHO)] can promote gastric stem cell–like properties by altering the microenvironment of the gastric mucosa (277), such as promoting the migration of myeloid cell differentiation factor Schlafen 4^+^ (SLFN4^+^) MDSCs into gastric metaplasia (278), enhancing the infiltration of MDSCs in the tumor microenvironment, and increasing their number (279), which are beneficial to the generation, proliferation, and survival of GC CSCs (280). Therefore, reducing the number of MDSCs will attenuate the generation of GC stem cells, and targeting CSCs can also play an anti-tumor effect by indirectly reducing MDSCs.

Treatments to inhibit the immunosuppressive function of MDSCs include phosphodiesterase-5 inhibitors (e.g., sildenafil and tadalafil) (281, 282), cyclooxygenase-2 inhibitors (COX-2), triterpenoids, and some targeted drugs (283). Phosphodiesterase-5 inhibitors can reduce the function of MDSCs by downregulating the expression of ARG1, IL4Ra, and ROS (282), which have shown positive results in patients with head and neck squamous cell carcinoma and melanoma in clinical trials (284, 285). The function of MDSCs has been suppressed by celecoxib as an immunomodulator of targeting COX-2 (286). In addition, triterpenoids can inhibit the suppression of effector T cells by MDSCs-mediated and have shown promising anticancer results in phase I clinical trials (287). The targeted drug tyrosine kinase inhibitor sunitinib can modulate anti-tumor immunity by reversing the immunosuppression mediated by MDSCs (288). In addition to mediating MDSCs migration, the CCR1 and CCR5 silenced *in vivo* can also lead to repolarization of MDSCs into tumor-killing neutrophils thus playing an anti-tumor effect (256).

MDSCs play a crucial role in promoting tumor progression and metastasis and generating immunosuppressive TME. The efficacy is limited in targeting MDSC treatments as monotherapy although they have seen efficacy in preclinical studies, whereas, combining with ICIs, they have seen synergistic effects in animal tumor models and have entered the clinical trials stage (244). Therefore, the anti-tumor efficacy may be increased by combining anti-MDSCs therapy with other anti-tumor means or combining multiple anti-MDSCs therapies, which is a promising therapeutic strategy.

## TANs-targeted therapy

Neutrophils can also be targets for anticancer therapy and strategies mainly include suppressing neutrophil immunosuppression by altering neutrophil recruitment and migration, depleting neutrophils at tumor sites, increasing neutrophil anti-tumor activity, and altering neutrophil polarity.

Neutrophils in the BM are released and migrated to the tumor microenvironment under the stimulation of mediators such as granulocyte CSF (G-CSF), granulocyte-macrophage CSF (GM-CSF), and chemokines such as CXC and CCL3 (289). TANs exhibits two subtypes under the action of cytokines in the tumor microenvironment: N1 TANs with anti-tumor effect and N2 TANs with tumor support activity (290). N1 TANs can directly kill tumor cells by releasing ROS and reactive nitrogen species (RNS), which can also promote the activation of T cells and the recruitment of M1 TAMs, whereas N2 TANs inhibits the function of NK cells and recruits M2 TAMs and T_regs_. It can also release matrix metalloproteinase 9 (MMP9) to promote angiogenesis and the spread of tumor cells (291). The N2 TANs phenotype increased in the TME since the high expression of TGF-β in the tumor microenvironment (292). Neutrophils are highly enriched and can enhance GC cell migration, invasion, and epithelial-mesenchymal transition (EMT) by secreting IL-17a in GC (293). The infiltration of neutrophils is closely related to the development of metachronous GC after endoscopic submucosal dissection (ESD) (294). Furthermore, the high levels of TANs are associated with disease progression and poor prognosis in GC (295). TANs are an independent risk factor for LNM in patients with early GC (EGC) (296). In addition, one study established a method to measure N2 TANs (cN2: CD15 minus CD66) and the results showed that cN2 TANs were closely associated with clinicopathological factors such as T stage, lymphatic, and perineural invasion in GC and were an independent marker of poor prognosis in DFS and OS (290). In addition, treatments can affect TANs, and the density of TANs decreased in tumor tissues of GC after neoadjuvant therapy compared with untreated GC (297).

The number of TANs can be reduced by decreasing neutrophil migration and recruitment to tumor sites in the tumor microenvironment. G-CSF can support tumor progression by mobilizing TANs (298), and neutralizing G-CSF or neutralizing IL-17, the upstream regulator of G-CSF can prevent neutrophil accumulation and downregulate the T-cell inhibitory phenotype of neutrophils (299). Furthermore, the CXCL/CXCR1/2 signaling axis is critical for neutrophil recruitment (300) and inhibition of CXCR1/2 signaling can reduce neutrophil recruitment (301); in addition, CXCR2 blockade as a single drug can prevent TAN accumulation and reduce tumor burden in tumor-bearing mice and can also enhance the efficacy of chemotherapy and immunotherapy (302, 303). The chemokine CXCL8 and the chemokine receptor CXCR4 are also involved in the recruitment of neutrophils in the tumor microenvironment. Inhibiting CXCL8 or blocking CXCR4 can inhibit the infiltration of TANs in TME (304–307). IL-6 can also attract TANs to the tumor environment and can attenuate and reverse the pro-inflammatory effects of neutrophils in the tumor microenvironment thus leading to immune-killing resistance. Therefore, immunotherapy targeting IL-6 is a potential target for tumor treatment *via* TANs (308). In addition, some targeted drugs such as ALK inhibitor lorlatinib and c-Met inhibitor capmatinib can also reduce TANs by inhibiting the entry of neutrophils in the BM into circulation (309, 310).

Depletion of TANs at tumor sites or neutrophil subsets with tumor-promoting functions is also a therapeutic strategy. The morphology and functions of polymorphonuclear MDSCs (PMN-MDSCs) are very similar to N2 TANs. The research showed that targeting TRAIL-R2 resulted in the elimination of different populations of MDSCs such as PMN-MDSCs and eMDSCs, without affecting mature myeloid or lymphoid cells (311). Splenectomy or the tyrosine kinase inhibitor sorafenib (low dose) can also attenuate or inhibit the inhibitory effect of tumor PMN-MDSCs on T-cell proliferation and cytotoxic activity (312).

In addition, it can also treat tumors by increasing the anti-tumor ability of neutrophils, and targeting Fc receptors on neutrophils can play an anti-tumor effect through antigen-dependent cytotoxicity (ADCC). Neutrophil-dependent ability induced by different tumor-associated antigens to kill tumor cells had been confirmed in extensive preclinical experiments in CD89 transgenic mice (including breast, colon, renal cell carcinoma, and T- and B-cell lymphoma) (313). Blockade of the Fas ligands that are upregulated by PMN-MDSCs can improve the anti-tumor efficacy of adoptive T-cell therapy in the TiRP melanoma model and improve the efficacy of checkpoint blockade in transplanted tumors (314).

Altering neutrophil polarization is also a therapeutic strategy, such that the immunosuppressive cytokine TGF-β can differentiate neutrophils into the N2 phenotype, and blocking TGF-β using the TGF-β inhibitor SM16 resulted in the accumulation of N1 TANs (312). Furthermore, type I IFN also can polarize TANs into the N1 phenotype in mouse tumor models, and similar changes were observed in melanoma patients with treated IFN-β (315).

A variety of therapeutic strategies targeting neutrophils are being carried out, but most of them are in the preclinical research stage; therefore, further validation of data from clinical trials is required.

## Therapies targeting B cells

**Figure 4:**
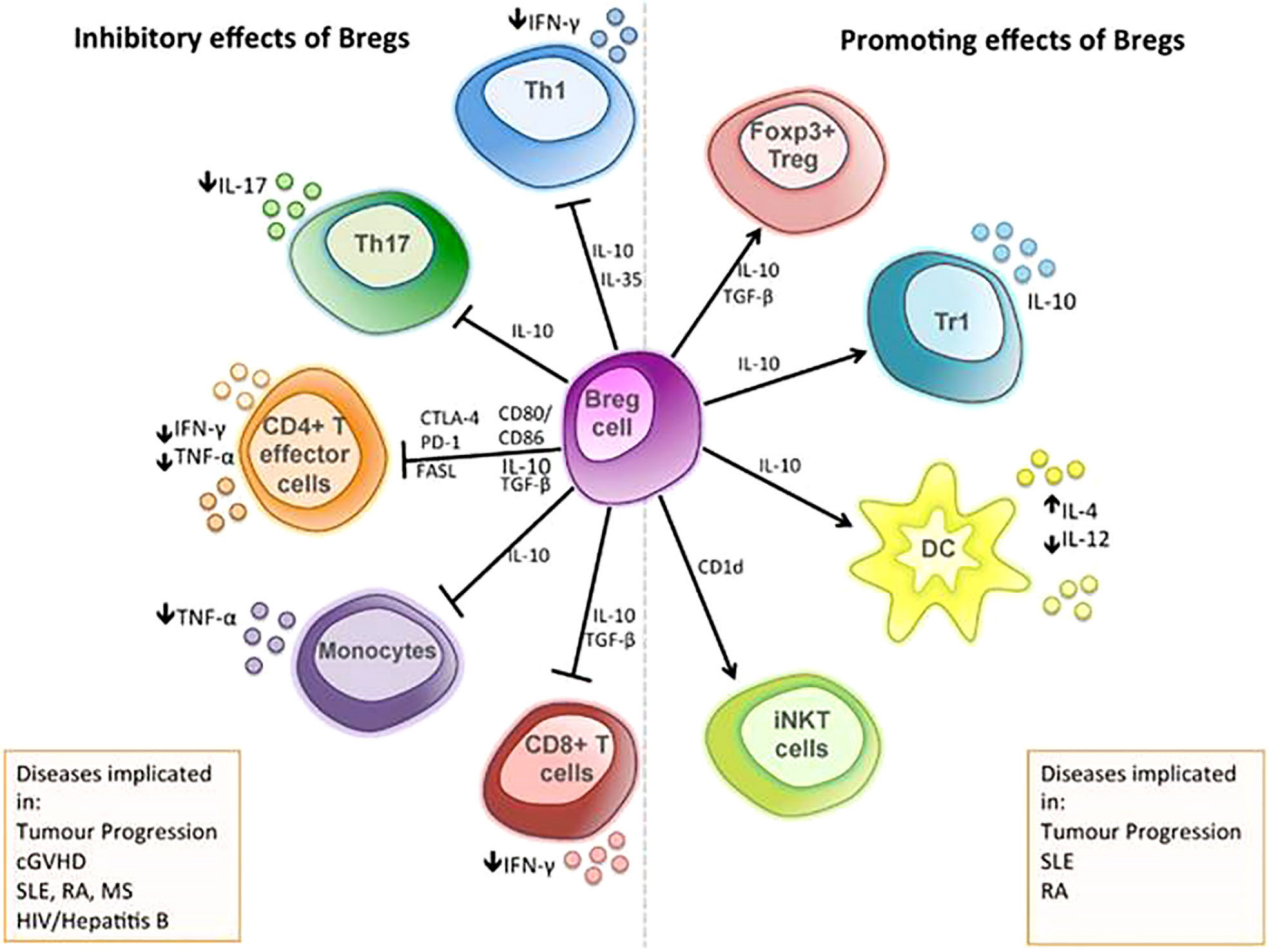
The immunosuppressive mechanisms of B_regs_: the functional mechanisms of B_regs_ are mediated through the release of soluble factors, such as IL-10, TGF-β, and IL-35, and through direct cell-cell contact *via* co-stimulatory molecules, including the inhibition of T-cell differentiation into type 1 T helper (T_H_1) cell and type 17 T helper (T_H_17) cell; inhibit the production of pro-inflammatory cytokine by CD4^+^ effector T cells; inhibit the production of TNF-α by monocytes; and inhibit the responses of cytotoxic CD8^+^ T cell. B_regs_ can initiate apoptosis of effector T cells through the expression of FASL and can also promote the differentiation of Foxp3^+^ T cells and type 1 regulatory T (Tr1) cell, alter cytokine production by dendritic cells, and support the maintenance of iNKT cells, which may have regulatory functions (318).

B cells can not only participate in the humoral immune response by producing antibodies and cytokines but also have a role in antigen presentation and immune regulation. In tumors, B cells are mainly concentrated in the tumor margin and the lymph nodes close to the tumor (316). In addition, B cells infiltrating the tumor margin differentiate into B_regs_ under the action of growth factors and different signaling pathways, which can support tumor growth by suppressing anti-tumor responses through producing anti-inflammatory cytokines and expressing inhibitory molecules (317) (**Figure 4**). For example, it can inhibit T_H_17, T_H_1, and CD8^+^ T-cell responses; inhibit CD4^+^ T-cell proliferation and induce their death (319); inhibit the production of IFN-γ and TNF-α (320); promote T_regs_ expansion (320) and increase the expression of CTLA4 on T_regs_ (321); secrete TGF-β, IL-10, and IL-35 (322); and affect the balance of T_H_1/T_H_2 (323). In addition, B_regs_ also express PD-1 and PD-L1 (322). In GC, B_regs_ are significantly increased in tumor tissues compared with surrounding tissues (324, 325), and the frequency of which in peripheral blood is significantly higher than that in the healthy control group (326). Studies have shown that B_regs_ were significantly associated with poor prognosis in GC patients. The 5-year OS rate in B_reg_^Low^ GC patients was significantly better than that in B_reg_^High^ patients (326). Therefore, reducing B_regs_ can help increase the anti-tumor response based on B_regs_-mediated immune escape. The treatments can take measures such as reducing the number of B_regs_ and reversing B cell–mediated immunosuppression. The number of B_regs_ can be reduced by proteasome inhibitor bortezomib, MEK inhibitor cobimetinib, and CD22 antibodies (327–329). Anti–IL-10 antibodies can inhibit the secretion of IFN-γ and TNF-α by B_regs_ (324). In addition, reversing B cell–mediated immunosuppression is also a therapeutic strategy. For example, the widely used ICI PD-1 antibody (nivolumab or pidilizumab) has been shown to reverse B cell–mediated immunosuppression (318). Above all, therapeutic approaches targeting B_regs_ have achieved some success but they are mostly based on preclinical studies with limited data and needed more research to support.

## Conclusion and prospects

The incidence of GC remains high in China, South Korea, Japan, and other Asian countries, and most patients are diagnosed in the middle or late stages because early cancer screening has not been popularized in some areas and the symptoms are not typical. The OS has not been significantly improved in the treatment of advanced GC after years of effort. However, the emergence of immunotherapy has brought hope to these patients. This review introduces the effects of various immune cells on the occurrence, development and prognosis of GC in the tumor microenvironment. Among them, tumor-infiltrating DCs, NK, M1 TAMs, and N1 TANs are beneficial to anti-tumor immunity and are associated with better prognosis in GC, whereas M2 TAMs, MDSCs, N2 TANs, eT_regs_, and B_regs_ and the expressions of PD-1, CTLA-4, LAG-3, TIM-3, and TIGIT on various immune cells can promote immune escape and are associated with poor prognosis in GC. Based on the importance of tumor-infiltrating immune cells to patient survival, the database Tumor Immunoassay Resource (TIMER) conducts a comprehensive analysis of tumor immunology and clinical and genomics data, which is used to estimate the abundance of six tumor-infiltrating immune cell (TIIC) subsets (B cells, CD4 T cells, CD8 T cells, macrophages, neutrophils, and DCs) to comprehensively study the molecular characterization of tumor-immune interactions. The TIMER database consists of six functional modules, including the association of TIIC abundance with gene expression (Gene), OS (Survival), somatic mutations (Mutation), and DNA somatic copy number alterations (SCNA), as well as analysis of differential gene expression (DiffExp) and gene-gene correlations (Correlation) (330). For example, using the TIMER database found that TGF-β2 had the ability to regulate immune cell recruitment and activation in gastric adenocarcinoma (STAD), and it might be an important regulator of immune cell infiltration and a valuable prognostic biomarker in GC patients (331). The expression of collagen family members positively correlated with infiltration of macrophages and expression of M2 macrophage markers, and with significant effects on tumor immunology (332). In addition, the expression of long non-coding RNA (lncRNA) target genes NOX4, COL8A1, and CHST1 was positively correlated with the degree of infiltration of CD8^+^ T cells, CD4^+^ T cells, macrophages, neutrophils, and DCs in the immune microenvironment, and these lncRNA target genes may be involved in the formation of the tumor immune microenvironment (333). Applying the TIMER tool analysis also showed that the expression of proteasome 26*S* subunit and ATPase gene (PSMC) family members was correlated with tumor purity, immune infiltration profile, and markers of different types of immune cells, which may become a new and important prognostic biomarker for tumor development (334). Therefore, the application of the TIMER database can help to discover new therapeutic targets and new immune evasion mechanisms.

The number of anti-tumor immune cells is reduced and their functions are suppressed in the tumor microenvironment, whereas the number of immunosuppressive cells is increased. Increasing the number or enhancing the function of anti-tumor immune cells, or reducing the number or weakening the function of immunosuppressive cells can restore anti-tumor immunity. Based on this, many studies have been carried out to target immune cells as a strategy for treating tumors, such as the ICIs PD-1/PD-L1 antibody and CTLA-4 antibody, which are widely used in clinical practice. The ICIs expressed on a variety of immune cells but mainly restore the anti-tumor activity of T cells. In addition, T cells also express LAG-3, TIM-3, TIGIT, and other receptors, and these immunosuppressive receptors are potential therapeutic targets and have seen anti-tumor efficacy in preclinical studies. ACTs are also promising treatment methods based on T-cell immunotherapy, such as CAR-T, TIL, and CIK. Among them, CAR-T has achieved amazing efficacy in hematological tumors, and CAR-T targeting CLDN18.2 has seen better ORR and DCR in the treatment of GC. However, the wide clinical application is limited due to the complicated preparation process and high cost of ACT, such as TIL and CIK. DC-CIK also belongs to ACT in DC-based therapy. Another DC-based treatment is the cancer vaccine, and these two treatment strategies can see anti-tumor effects when combined with other treatments such as chemotherapy. The anti-tumor activity of immune cells can be restored by reducing immunosuppressive cells or inhibiting the activity of immunosuppressive cells, such as M2 TAMs, MDSCs, N2 TANs, eT_regs_, B_regs_, and the reduction of immunosuppressive cells can be achieved by blocking the migration of immunosuppressive cells into the TME. For example, CSF-1/CSF-1R mediates the chemotaxis of TAMs, TANs, and MDSCs; CXCR1/2 mediates the chemotaxis of TANs and MDSCs; CXCR4 participates in the chemotaxis of TAMs, TANs, and T_regs_; and CCL5-CCR5 participates in the chemotaxis of TAMs and MDSCs. Blocking the interaction of these chemokines with their receptors can reduce the migration of immunosuppressive cells to the TME. In addition, “reprogramming” immune cells from tumor support cells to tumor killer cells can also increase anti-tumor activity. For example, M1 TAMs and N1 TANs can inhibit and kill tumors, whereas M2 TAMs and N2 TANs promote tumor progression and tumor angiogenesis. Using TGF-β inhibitors or type I IFN can polarize TANs to N1 phenotype. Meanwhile, TLR agonists, CD40 and CD47 antibodies, CSF-1/CSF-1R inhibitors or neutralizing antibodies, and TGF-β, HDAC, and PI3Kγ inhibitors all can reprogram TAMs to M1 TAMs. These reprogramming therapeutic strategies have seen anti-tumor efficacy in preclinical tumor models. In addition, CAR-NK and CAR-M have been developed and applied for anti-tumor therapy based on the success of CAR-T in hematological tumors, and both have entered the clinical trial stage. Among them, the activity of CAR-NK is similar to CAR-T cells and safer than that and does not have strict HLA-matching requirements, so it is a potential strategy for the treatment of GC. The treatment strategies to increase anti-tumor immune cells or their functions are ACTs which was mentioned before, such as CAR-T, CAR-NK, CAR-M, TIL, CIK, DC-CIK, etc.

There are many strategies for targeting immune cells to treat tumors, among which the treatment strategies targeting T cells have the most significant effect and the fastest development and have shown good clinical efficacy in hematological tumors and various solid tumors, such as ICIs PD-1/PD-L1, CTLA-4, and CAR-T in adoptive immune cell therapy. However, these immunotherapies also have limitations in the treatment of GC. For example, the aforementioned anti–PD-1/PD-L1 antibodies and anti–CTLA-4 antibodies have failed to improve OS and PFS in patients compared with chemotherapy in some trials. Although positive results have been obtained in some clinical trials, most of the benefited populations are only those with high PD-L1 CPS score, MSH-H, or high TMB, but unfortunately these populations do not account for much of advanced GC. ICI can also cause the occurrence of fatal toxic events. According to statistics, the toxicity-related mortality rates were 0.36% (anti–PD-1), 0.38% (anti–PD-L1), and 1.08% (anti–CTLA-4). A total of 613 fatal ICI toxicity events were reported in the WHO pharmacovigilance database (Vigilyze) from 2009 to January 2018, of which anti–PD-1/PD-L1-related deaths were usually from pneumonia (35%), followed by hepatitis (22%) and neurotoxicity (15%), whereas anti–CTLA-4–related deaths were mostly due to colitis (70%) (335). The CAR-T cell therapy also has limitations in the treatment of GC, including life-threatening–related toxicity such as cytokine release syndrome (CRS), hemophagocytic lymphohistiocytosis and/or macrophage activation syndrome (MAS), and immune effector cell-associated neurotoxicity syndrome (ICANS). Moreover, the efficacy of CAR-T in the treatment of GC is still limited. In addition, there is still antigen escape and the immunosuppressive tumor microenvironment and physical tumor barriers such as the tumor stroma limit the penetration and mobility of CAR-T cells (336). Other strategies for targeting immune cells to treat tumors have shown efficacy in preclinical animal models but have not been widely used in the clinic, which is partly because some treatment strategies are in clinical trials, and results have not yet been published. Another part of the reason may be related to the insignificant efficacy. For example, the two cancer treatment strategies based on DCs (vaccine and DC-CIK) are only effective when they are combined with other treatment options such as chemotherapy. The widely used ICIs are only effective for some tumor patients, whereas other patients will develop primary or secondary drug resistance. The reason for the poor efficacy of immunotherapy may be related to the complex microenvironment in which the tumor is located. The interaction of tumor cells, immune cells, and stromal cells in the tumor microenvironment constitutes a huge immune suppression network that leads to tumor immune escape, but the current immunotherapy only targets one type of cells or a certain target on a type of cells, whereas the immunosuppressive environment is composed of multiple cells and multiple targets. For example, more than 10 kinds of immunosuppressive receptors expressed on T cells have been confirmed, and there are many unexplored inhibitory receptors. The development of multi-cell and multi-target therapeutic strategies may be the future development direction. Based on this, multi-target combination strategies have been used for tumor treatment, such as PD-1/PD-L1 inhibitor combined with CTLA-4 inhibitor and PD-1 inhibitor combined with anti–LAG-3 (74, 337). At the same time, the PD-1/LAG-3 dual antibody has been developed and has entered the clinical trial stage (68). In addition, therapeutic strategies targeting immune cells have also achieved good results in combination with other treatments, such as combination with chemotherapy drugs. It has been confirmed that drug therapy plays a role in remodeling the tumor immune microenvironment, such as decreased density of CD8^+^ cells and increased density of FoxP3^+^ cells and B cells (CD20^+^) in PTs after NAC, but PD-L1 expression did not change (338). In addition, PD-L1 expression and CD8^+^ T-cell infiltration were increased in the tumor microenvironment after treatment with the targeted drug anti-VEGFR2 antibody RAM in GC, and PD-1 expression in eT_regs_ cells and CD8^+^ T cells was significantly reduced in TILs (136). However, this multi-target or targeting multi-cell or combined with other therapies to treat tumors may bring more toxic side effects while increasing the anti-tumor efficacy. Therefore, there is a long way to go for anti-tumor therapy targeting immune cells to achieve synergy and detoxification.

## Author contributions

YZ planned, designed, and wrote the majority of the manuscript. MS guided the project and wrote the manuscript. YB and YL planned and guided the project and wrote the manuscript. All authors contributed to the article and approved the submitted version.

## Conflict of Interest

The authors declare that the research was conducted in the absence of any commercial or financial relationships that could be construed as a potential conflict of interest.

## Publisher’s note

All claims expressed in this article are solely those of the authors and do not necessarily represent those of their affiliated organizations, or those of the publisher, the editors and the reviewers. Any product that may be evaluated in this article, or claim that may be made by its manufacturer, is not guaranteed or endorsed by the publisher.

## Glossary

**Table:**

GC	Gastric cancer
TME	Tumor microenvironment
DCs	Dendritic cells
NK	Natural killer
TAMs	Tumor-associated macrophages
TANs	Tumor-associated neutrophils
MDSCs	Myeloid-derived suppressor cells
Tregs	Regulatory T cells
TH	T helper
eTregs	Effector Tregs
Bregs	Regulatory B cells
APC	Antigen-presenting cells
ICI	Immune checkpoint inhibitors
PD-1/PD-L1	Programmed cell death 1/programmed death-ligand
mAbs	Monoclonal antibodies
CTLA-4	Cytotoxic T lymphocyte–associated protein 4
TMB	Tumor mutational burden
EBV	Epstein–Barr virus
CAR-T	Chimeric antigen receptor T
OS	Overall survival
DFS	Disease free survival
PFS	Progression-Free-Survival
ORR	Objective response rate
DCR	Disease control rate
MSI-H	Microsatellite instability-high
LAG-3	Lymphocyte activation gene 3
TIM-3	T-cell immunoglobulin and mucin domain-3
TIGIT	T cell immunoreceptor with immunoglobulin and ITIM domain
ACT	Adoptive cell therapy
MSLN	Mesothelin
PSCA	Prostate Stem Cell Antigen
HER2	Human epidermal growth factor receptor 2
CEA	Carcinoembryonic antigen
TIL	Tumor-infiltrating lymphocyte
CIK	Cytokine-induced killer cells
ICOS	Inducible T-cel l costimulator
GITR	Glucocorticoid-induced TNFR-related protein
OX40	Tumor necrosis factor receptor 4
VEGFR2	Vascular endothelial growth factor receptor-2
CCL/CXCL	Chemokine
CCR/CXCR	Chemokine receptors
FOXP3	Forkhead box P3
DC-CIK	Dendritic Cell -cytokine Induced Killer Cell
IL	Interleukin
ADCC	Antibody-dependent cell-mediated cytotoxicity
KIR	Killer immunoglobulin receptor
PI3K	Phosphoinositide 3- kinase
CAR-NK	Chimeric antigen receptor natural killer
CAR-M	Chimeric antigen receptor macrophage
CSF-1/CSF-1R	Colony-stimulating factor 1/Colony-stimulating factor 1 receptor
TLR	Toll-like receptors
HDAC	Histone deacetylase
ATRA	All-trans retinoic acid
COX-2	Cyclooxygenase 2
GCSF	Granulocyte colony-stimulating factor
ARG1	Arginase I
iNOS	Inducible nitric oxide synthase
NO	Nitric oxide
MMPs	Matrix metalloproteinases
ROS	Reactive oxygen species
PMNMDSC	Polymorphonucler myeloid-derived suppressor cell
GMDSCs	Granulocyte-like myeloid-derived suppressor cells
CSCs	Cancer stem cells
MSCs	Mesenchymal stem cells
TH	Helper T cells
IFN	Interferon
TNF	Tumor necrosis factor
TGF	Transforming growth factor
CapeOx	Capecitabine plus oxaliplatin
FOLFOX	Leucovorin calcium plus fluorouracil plus oxaliplatin
